# Supplementary material for: Cytogenetic Effects in Children Exposed to Air Pollutants: A Systematic Review and Meta-Analysis
Source: Int J Environ Res Public Health. 2022 May 31;19(11):6736. doi: 10.3390/ijerph19116736 (PMC9180689; doi:10.3390/ijerph19116736)
Supplement: Supplementary file 1 [file ijerph-19-06736-s001.zip › ijerph-1685097-supplementary.pdf]

Table S1. Search strategy in PubMed/MEDLINE.

SET	PubMed/MEDLINE
1	micronucleu*[Title/Abstract]
2	micronucleu*[Text Word]
3	assay, micronucleus[MeSH Terms]
4	Sets 1-3 were combined with "OR"
5	child*[Title/Abstract]
6	child*[Text Word]
7	children[MeSH Term]
8	Sets 5-7 were combined with "OR"
9	air pollution[Title/Abstract]
10	air pollution[Text Word]
11	air pollution[MeSH Terms]
12	air pollution, indoor[MeSH Terms]
13	air pollutants[Title/Abstract]
14	air pollutants[Text Word]
15	air pollutants[MeSH Term]
16	air pollutants, environmental [MeSH Terms]
17	air pollutants, particulate [MeSH Terms]
18	Sets 9-17 were combined with "OR"
19	Sets 4, 8 and 18 were combined with "AND"

Table S2. Description of inclusion and exclusion criteria according to population (P), exposure (E), outcomes (O) and study design (S).

Search strategy	Details
Inclusion criteria	P: healthy children in young populations including age range 5–12
	E: exposure to environmental pollutants
	O: micronuclei in donors' cells
	S: cross-sectional studies
Exclusion criteria	P: subjects > 16 years old
	E: absence of two differently exposed groups
	O: other cytogenetic alterations
	S: opinion papers, reviews and meta-analyses, commentary, letters, protocols, thesis, conference papers, note, book chapter, clinical trials
Language filter	English
Time filter	No time filter
Databases	PubMed/MEDLINE; Scopus; Web of Science

Table S3. Main demographic characteristics and exposure patterns of the studied populations, studies reported in chronological order.

Author/s, year [ref.]	No. of recruited children (gender)[a]	Age (years)	Exposure
Sabah, 2021 [83]	E: 100 (67 M; 33 F)<br>NE: 100 (69 M; 31 F)	Overall: 6–8 (range)<br>- 6 years: 24 E / 25 NE<br>- 7 years: 38 E / 35 NE<br>- 8 years: 38 E / 40 NE	E: Living in the proximity (< 5 km) from an oil field.<br>NE: Living far (≥ 40 km) from an oil field.
Lemos et al., 2020 [78]	PBL - E: 28<br>NE: 8<br>BMC - E: 29<br>NE: 21	Overall: 5–12 (range)<br>8.35 ± 1.82 (mean)	E: $PM_{2.5}$ (13.9 ± 14.2 μg/m^3), PAHs (5.8 ng/m^3).<br>NE: PM_{10} (22.1 ± 20.2 μg/m^3); PAHs (8.4 ng/m^3).
Panico et al., 2020 [73]	E: 206 (97 M, 109 F)<br>NE: 256 (143 M, 113 F)	Overall: 6–8 (range)<br>E: 7.68 ± 0.80<br>NE: n.a. (similar, $p > 0.05$)	E: $PM_{0.5}$ (9.3 ± 2.6 μg/m^3); PM_{10} (22.2 ± 6.8 μg/m^3).<br>NE: $PM_{0.5}$ (3.2 ± 1.4 μg/m^3); PM_{10} (11.6 ± 3.9 μg/m^3).
Villarini et al., 2018 [74]	E: 1,046 (winter) (526 M; 520 F)<br>NE: 1,046 (late-spring) (526 M; 520 F)	Overall: 6–8 (range)<br>6.83 ± 0.90 (mean ± SD)	E: Five Italian towns in winter.<br>NE: Five Italian towns in summer (late-spring).
de Carvalho Cavalcante et al., 2017 [79]	E: 19 (12 M; 7 F)<br>NE: 24 (11 M; 13 F)	Overall: 10–14 (range)	E: School A, Vehicular Traffic Number = 1628.6.<br>NE: School B, Vehicular Traffic Number = 194.
Coronas et al., 2016 [80]	PBL - E: 41<br>NE: 17<br>BMC - E: 38<br>NE: 19	Overall: 5–12 (range)<br>8.5 (mean)	E: Living in the area adjacent to a wood treatment plant (risk area); $PM_{2.5}$ (μg/m^3):<br>- October 25.2<br>- December 35.5<br>- January 8.7 / 1.3<br>- February 8.9<br>NE: Living distant from a wood treatment plant (reference area); $PM_{2.5}$ (μg/m^3):<br>- October 33.3<br>- December 10.0<br>- January 19.9 / 39.0<br>- February 16.0

Table S3. Continued.

Author/s, year [ref.]	No. of recruited children (gender)[a]	Age (years)	Exposure
Mørck et al., 2016 [75]	E: 52 (23 M; 29 F)<br>NE: 48 (24 M; 24 F)	Overall. 9 ± 1.7	E: Urban area. Busy road within 50 m of home (%): 29<br>- NO_2: 17.1 ± 4.3 μg/m^3<br>- $PM_{2.5}$: 10.8 ± 0.4 μg/m^3<br>- PM_{10}: 13.2 ± 0.9 μg/m^3<br>- NO_2 above p75 (%): 52<br>- High traffic (%): 50<br>NE: Rural area. Busy road within 50 m of home (%): 15<br>NO_2: 10.3 ± 1.0 μg/m^3<br>$PM_{2.5}$: 10.0 ± 0.1 μg/m^3<br>PM_{10}: 12.2 ± 0.2 μg/m^3<br>NO_2 above p75 (%): 0<br>High traffic (%): 8
da Silveira Fleck et al., 2014 [81]	E: 33 (9 M; 24 F)<br>NE: 34 (15 M; 19 F)	E: 13.7 ± 1.1<br>NE: 13.1 ± 0.9	E: high population density.<br>Summer<br>NO_2: 42.4 ± 5.0 μg/m^3<br>O_3: 43.2 ± 8.1 μg/m^3<br>Winter<br>NO_2: 42.1 ± 4.1 μg/m^3<br>O_3: 35.9 ± 12.9 μg/m^3<br>Traffic: 5060 vehicles/h<br>NE: low population density.<br>Summer<br>NO_2: 13.9 ± 3.0 μg/m^3<br>O_3: 34.3 ± 9.6 μg/m^3<br>Winter<br>NO_2: 17.1 ± 7.0 μg/m^3<br>O_3: 23.7 ± 10.1 μg/m^3<br>Traffic: 1607 vehicles/h

Table S3. Continued.

Author/s, year [ref.]	No. of recruited children (gender)[a]	Age (years)	Exposure
Ceretti et al., 2014 [76]	E: 97<br>NE: 25	Overall: 3–6 (range)<br>4.35 ± 0.84 (mean ± SD)	E: Heavy traffic (residence area)<br>NE: Very light traffic (residence area)
Demircigil et al., 2013 [84]	E: 93 (winter) (43 M; 50 F)<br>NE: 93 (summer) (43 M; 50 F)	Overall: 9–13 (range)	E: Winter sampling<br>Suburban<br>NO_2: 7 μg/m^3<br>SO_2: 28 μg/m^3<br>O_3: 90 μg/m^3<br>PM_{10} 47.6 ± 17.1 μg/m^3<br>Urban-traffic<br>NO_2: 39 μg/m^3<br>SO_2: 64 μg/m^3<br>O_3: 36 μg/m^3<br>PM_{10} 45.7 ± 17.2 μg/m^3<br>NE: Summer sampling<br>Suburban<br>NO_2: 5 μg/m^3<br>SO_2: 17 μg/m^3<br>O_3: 122 μg/m^3<br>$PM_{2.5}$: 22.6 ± 7.13 μg/m^3<br>PM_{10}: 85.9 ± 22.60 μg/m^3<br>Urban-traffic<br>NO_2: 12 μg/m^3<br>SO_2: 10 μg/m^3<br>O_3: 79 μg/m^3<br>$PM_{2.5}$: 25.2 ± 6.79 μg/m^3<br>PM_{10}: 75.0 ± 17.34 μg/m^3

Table S3. Continued.

**Author/s, year [ref.]**	**No. of recruited children (gender)**[a]	**Age (years)**	**Exposure**
Sisenando et al., 2012 [82]	E: 245 (110 M; 135 F)<br>NE: 128 (54 M; 74 F)	Overall: 6–16 (range)<br>≤ 7 years: 78<br>8–9 years: 110<br>10–11 years: 119<br>12–13 years: 133<br>≥ 14 years: 134	E: $PM_{2.5}$: 21 μg/m^3 (2008)<br>NE: $PM_{2.5}$: 10 μg/m^3 (2008)
Pedersen et al., 2006 [77]	E: 23 (11 M; 12 F)<br>NE: 24 (13 M; 11 F)	Overall: 5–11 (range)<br>Younger children (5–7 years old): 6.48 ± 0.54 E / 6.24 ± 0.47 NE<br>Older children (7–11 years old): 9.31 ± 1.22 E / 9.43 ± 1.20 NE	E (Teplice—mining area):<br>airborne ultrafine particles (UFP): 15,000 ± 1900 UFP/mL<br>$PM_{2.5}$: 120 ± 1500 μg/m^3<br>PM_{10}: 238.6 μg/m^3<br>PAHs: 8.01 ng/m^3 (February 2004)<br>NE (Prachatice—less polluted area):<br>UFP: 13,000 ± 1900 UFP/mL<br>$PM_{2.5}$: 46 ± 2000 μg/m^3<br>PM_{10}: 120 μg/m^3<br>PAHs: 4.54 ng/m^3 (February 2004)
Lahiri et al., 2000 [85]	E: 153 (81 M; 72 F)<br>NE: 116 (61 M; 55 F)	Overall: 6–17 (range)	E: Living in a highly urbanized area.<br>NE: Living in a rural area.

[a] BMC, buccal mucosa cells; PBL, peripheral blood lymphocytes; WB, whole blood/leukocytes.
